# What is real change in submaximal cardiorespiratory fitness in older adults? Retrospective analysis of a clinical trial

**DOI:** 10.1186/s40798-022-00447-6

**Published:** 2022-04-28

**Authors:** Michelle Hall, Yuri Lopes Lima, Zoya Huschtscha, Fiona Dobson, Ricardo J. S. Costa

**Affiliations:** 1 Centre for Health, Exercise and Sports Medicine, Department of Physiotherapy, School of Health Sciences, The University of Melbourne, Melbourne, VIC 3010 Australia; 2 Department of Nutrition Dietetics & Food, Monash University, Notting Hill, VIC 3168 Australia

**Keywords:** Test–retest reliability, Aerobic economy, Cycle ergometer, Oxygen uptake, Respiratory exchange ratio

## Abstract

**Objective:**

To assess the test–retest reliability of submaximal cardiorespiratory fitness in healthy active older adults.

**Methods:**

This was a retrospective analysis of 41 adults enrolled in a clinical trial [mean (sd) aged 59 yrs (7); 29% females; and body mass index 24.5 kg/m^2^ (3.3)]. Cardiorespiratory fitness was assessed using a cycle ergometer 6 weeks apart. The initial workload was 1 W per kilogram of free fat mass (W/kg FFM) and increased by 0.5 W/kg FFM every 3 min until participants could not maintain the speed at ≥ 60 rpm, they reached a rating of perceived exertion of 15–17, and/or obtained a respiratory exchange ratio (RER) of 1.000. Reliability of ${V}{\text{O}}_{2}$_,_ heart rate and RER was assessed for each workload, and for ${V}{\text{O}}_{2}$_,_ when RER reached 1.00. Reliability was examined as the intraclass correlation coefficient (ICC_(2,1)_), Bland–Altman plots, standard error of measurement (SEM and SEM%), and the minimal detectable change (MDC).

**Results:**

Test–retest agreement ranged between (ICC_(2,1)_ 0.44–0.84) with no discernible systematic differences between assessments. The SEM% for absolute and relative ${V}{\text{O}}_{2}$ ranged between 13.0 to 20.2%, and 13.8 to 26.3%, respectively_._ The MDC_90_% for absolute and relative ${V}{\text{O}}_{2}$ ranged between 30.4% to 47.1%, and 32.2% to 61.4%, respectively. The lowest SEMs% and MDCs% for both absolute and relative ${V}{\text{O}}_{2}$ were observed for workloads at 2.5 W kg/FFM (~ 13% and ~ 31%, respectively).

**Conclusions:**

Although at least modest relative reliability was consistently demonstrated, the smaller measurement error associated with absolute and relative ${V}{\text{O}}_{2}$ at 2.5 W kg/FFM may indirectly suggest that submaximal cardiorespiratory fitness can be monitored more confidently at higher workloads. Findings provide critical information to determine how much change is considered ‘real change’ in repeated measures of cardiorespiratory fitness using a submaximal graded exercise testing protocol in healthy active older adults.

**Supplementary Information:**

The online version contains supplementary material available at 10.1186/s40798-022-00447-6.

## Key points

- Measures of submaximal cardiorespiratory fitness are at least ‘modestly’ reliable over 6 weeks
- Lowest measurement errors are associated with submaximal $\dot{V}{\text{O}}_{2}$ at highest workloads
- Largest measurement errors are associated with submaximal $\dot{V}{\text{O}}_{2}$ when respiratory exchange ratio reaches 1.000

## Introduction

Cardiorespiratory fitness (CRF) is a predictor of cardiovascular disease and all-cause mortality [1]. Hence, improving cardiorespiratory fitness is the aim of many interventions. An incremental exercise test to volitional exhaustion (e.g. $\dot{V}{\text{O}}_{2\max }$ test) is considered the gold standard to assess CRF [2]. However, maximal exercise testing is typically limited to specific healthy athletic populations that habitually exert effort to maximal exhaustion. Moreover, the use of maximal exercise testing is limited in many older adults due to pain or fatigue rather than exertion and is contraindicated in older adults to undergo maximal exercise testing due to excessive cardiovascular strain [3]. Thus, submaximal exercise testing is the approach of choice by physical therapists for patients who are limited by pain or fatigue [3].

Two types of reliability include relative and absolute reliability. Relative reliability can be described using intraclass correlation coefficient (ICC) and is the extent to which individual maintain their position in a sample with repeated measurement [4]. Absolute reliability is the extent to which repeated measurements vary for individuals [4] and can be expressed as the absolute limits of agreement [4], the standard error of measurement (SEM) [4] and minimal detectable change (MDC). Absolute limits of agreement provide assessors with a range within which to expect differences between the test and retest for 95% of the population to lie between [5]. The SEM provides a range of values, which encompasses a true score on measure of interest, and can be expressed in the same units as the original measurement [6]. The MDC provides limits such that changes greater than the MDC can be interpreted as real change [7]. Knowledge of real change is particularly useful for monitoring and evaluating interventions [8]. This study aimed to assess test–retest reliability and real change in submaximal CRF over 6 weeks in older healthy active adults.

## Methods

Data from 41 participants (Table 1) enrolled in a randomised controlled trial evaluating the effects of a high-protein dairy milk beverage with or without progressive resistance training on anthropometric, power, strength, functional capacity, and pathophysiological variables of sarcopenia in healthy active older adults previously reported were used [9]. There were no within- or between-group effects on submaximal CRF [9]. The human ethics from the Monash University Research Ethics Committee approved the study in accordance with the standards of ethics outlined in the Declaration of Helsinki, and all participants provided written informed consent. The original clinical trial was registered with the Australian and New Zealand Clinical Trial Registry as ANZCT12618001088235.

**Table 1:** Participant characteristics presented as mean (SD) unless, otherwise stated

	n = 41
Age, year	59.1 (7.2)
Females, n (%)	12 (29%)
Height, m	1.72 (0.10)
Body weight, kg	73.2 (13.1)
Body mass index, kg/m^2^	24.5 (3.3)

### Participants

People were eligible if they were: (1) participating in ≥ 3 structured exercise sessions per week equivalent to ≥ 90 min/week. People were not eligible to participate if they: (1) had dairy protein allergy or lactose intolerances; (2) were currently taking protein supplements; (3) had any injuries preventing safe exercise; (4) had any surgery in the past 12 months; (5) had any cardiovascular-related complications; (6) had any thyroid conditions; (7) had weight loss of more than 5% body weight over last 6 months; (8) on medications that could interfere with muscle structure or function (e.g. corticosteroids); (9) undergoing immunosuppressive therapy or hormone replacement therapy; (10) consume more than two standard drinks of alcohol per day or 14 per week; (11) smoke; (12) had body mass index > 30 kg/m^2^; and (13) structured resistance exercise in the past 12 months.

### Descriptive measures

Age, biological sex, height, body mass (BM), body mass index (BMI) and submaximal cardiovascular fitness were assessed. BMI was measured (Seca 515 MBCA, Seca Group, Hamburg, Germany) to the nearest 0.1 kg, using standardised anthropometrical procedures. Submaximal cardiovascular fitness was assessed according to procedures described below.

### Procedures

Participants were asked to wear an activity monitor (ActiGraph wGT3X-BT, Actigraph, Pensacola, FL, USA) on their non-dominant wrist for 6 weeks after the first CRF assessment. Participants were asked to wear the activity monitor each day from waking up to going to bed and take the monitor off only when participating in aquatic pursuits. Throughout this period, participants were asked to engage in their usual lifestyle activities.

CRF was assessed on two separate testing sessions approximately 6 weeks apart using a cycle ergometer (Corival, Lode, Groningen, The Netherlands) and a metabolic cart (Vmax Encore Metabolic Cart, Carefusion, San Diego, CA). For each testing session, participants arrived at the laboratory between 7:00am and 9:00am in a fasted state and euhydrated state [plasma osmolality = 296 (5.4) mOsmol/kg (Osmomat 030, Gonotec, Berlin, Germany)]. All participants were instructed to avoid strenuous exercise for a 24-h period before attending laboratory assessments. Free fat mass (kg, FFM) was assessed by a single trained radiographer using a dual-energy X-ray absorptiometry (iDXA; Prodigy, GE Lunar, Madison, WI, USA) with analysis software 14.0. The initial workload was 1 W per kilogram of FFM (W kg/FFM) and increased by 0.5 W/kg FFM every 3 min until participants could not maintain the speed at ≥ 60 rpm, they reached a rating of perceived exertion (RPE) [10] of 15–17 and/or obtained a respiratory exchange ratio (RER) of 1.000. Heart rate (HR) (Polar, Electro, Kempele, Finland), RPE, $\dot{V}{\text{O}}_{2}$ and RER were measured in the last minute. Cardiorespiratory fitness was assessed as relative $\dot{V}{\text{O}}_{2}$ when RER reached one, in addition to HR, RPE, absolute and relative $\dot{V}{\text{O}}_{2}$, and RER for each workload.

### Data analysis

Data were checked for normal distribution using the Shapiro–Wilk test and visual inspection of Q–Q plots. Test–retest reliability was not determined for RPE, as it did not conform to normal distribution across workloads despite efforts to transform the data using various techniques (e.g. log transform, etc.). Paired t-test was used to determine whether there were significant difference for any of the measures between two time points. Bland–Altman plots were used to examine heteroskedasticity and systematic changes in the mean and illustrate absolute limits of agreement. Absolute limits of agreement were calculated as the mean difference $\times$ 1.96 (standard deviation). The degree of heteroskedasticity was also measured by calculated the Kendall’s tau correlation between the absolute differences and corresponding means. In the event of a statistically significant correlation coefficient at *p* < 0.05, the data were denoted as heteroskedastic, and data were subsequently transformed by logarithms to the base 10 and re-assessed for heteroskedasticity.

Agreement between measures (relative reliability) was calculated using intraclass correlation coefficient (ICC_2,1_) with 95% confidence intervals (CI) for a two-way random effects model and absolute agreement. Point estimates of the ICCs were interpreted as follows: excellent (0.75–1.00), modest (0.40–0.74), or poor (0–0.39) [11]. Measurement errors were evaluated using the standard error of measurement (SEM) and SEM%. The SEM was calculated as the square root of the mean square error term from the ANOVA, with 95% CI around SEM [6]. The SEM percentage (SEM%) was calculated by dividing the mean value and multiplying by 100. To estimate the smallest change that indicates a ‘real change’ in 90% of individuals, the MDC_90_ and MDC_90_% were calculated. The MDC_90_ was calculated as SEM $\times$ 1.65 (z score of 90% interval) $\times$ √2. The MDC_90_ percentage (MDC_90_%) was calculated by dividing the MDC by respective mean value and multiplying by 100. SPSS (version 24, SPSS Chicago, IL USA) was used to preform analyses.

## Results

There was no evidence of significant differences for any of the measures between the two time points (*p* > 0.05). Test–retest reliability data are presented in Table 2. Time spent in physical activity according to intensity did not significantly change in the 6-week study period compared to the 6 weeks prior to the first CRF assessment (Additional file 1), although time in very vigorous activity per day changed by 4 min (*p* = 0.05).

**Table 2:** Test–re-test reliability and measurement error rate estimates at baseline and 6 weeks

Workload	Parameter	Baseline mean (SD)	6-week mean (SD)	Mean difference (95% CI)	Tau-correlation (absolute difference v mean)	ICC_(2,1)_ (95% CI)	SEM (95% CI)	SEM %	MDC_90_ (%)
Original measurements									
1.0 W kg/FFM									
(n = 41)	$\dot{V}{\text{O}}_{2}$ (ml/kg/min)	12.68 (3.14)	12.47 (3.61)	0.21 (− 0.92, 1.34)	< 0.01	0.44 (0.16, 0.66)	2.54 (2.08, 3.94)	20.2	5.93 (47.1)
(n = 41)	$\dot{V}{\text{O}}_{2}$ (L/min)	0.87 (0.29)	0.84 (0.29)	0.03 (− 0.04, 0.10)	0.12	0.67 (0.45, 0.81)	0.17 (0.14, 0.21)	19.8	0.40 (46.1)
(n = 38)*	Heart rate (bpm)	89.11 (12.43)	86.97 (13.74)	2.13 (− 0.95, 5.21)	− 0.11	0.74 (0.56, 0.86)	6.67 (5.50, 8.62)	7.6	15.6 (17.7)
(n = 41)	RER	0.92 (0.08)	0.92 (0.08)	0.01 (− 0.02, 0.03)	0.13	0.57 (0.32, 0.75)	0.05 (0.04, 0.07)	5.4	0.12 (12.7)
1.5 W kg/FFM									
(n = 34)	$\dot{V}{\text{O}}_{2}$ (ml/kg/min)	16.06 (3.86)	16.28 (3.92)	− 0.21 (− 1.54, 1.11)	< 0.01	0.53 (0.23, 0.73)	2.69 (2.17, 3.54)	16.1	6.28 (37.6)
(n = 34)	$\dot{V}{\text{O}}_{2}$ (L/min)	1.11 (0.36)	1.12 (0.36)	− 0.01 (− 0.14, 0.12)	0.23	0.69 (0.46, 0.83)	0.20 (0.17, 0.27)	17.7	0.47 (41.3)
(n = 33)^*^	Heart rate (bpm)	98.45 (10.78)	97.91 (13.18)	0.54 (− 2.20, 3.29)	0.13	0.80 (0.63, 0.89)	5.48 (4.41, 7.25)	5.6	12.8 (13.0)
(n = 32)^**^	RER	0.95 (0.06)	0.95 (0.06)	− 0.00 (− 0.02, 0.02)	− 0.12	0.63 (0.37, 0.80)	0.03 (0.02, 0.05)	3.2	0.07 (7.4)
2.0 W kg/FFM									
(n = 25)	$\dot{V}{\text{O}}_{2}$ (ml/kg/min)	20.87 (4.84)	20.29 (5.04)	0.58 (− 1.16, 2.33)	0.15	0.64 (0.33, 0.82)	2.99 (2.34, 4.16)	14.5	6.98 (33.9)
(n = 25)	$\dot{V}{\text{O}}_{2}$ (L/min)	1.49 (0.51)	1.43 (0.48)	0.06 (− 0.09, 0.21)	0.23	0.74 (0.49, 0.87)	0.25 (0.20, 0.35)	17.1	0.58 (40.0)
(n = 25)	Heart rate (bpm)	109.88 (15.63)	109.88 (15.65)	0.00 (− 6.63, 6.63)	**0.28**	–	–	–	–
(n = 25)	RER	0.98 (0.07)	0.98 (0.07)	− 0.00 (− 0.03, 0.02)	0.08	0.57 (0.22, 0.78)	0.04 (0.03, 0.06)	4.1	0.09 (9.5)
2.5 W kg/FFM									
(n = 17)	$\dot{V}{\text{O}}_{2}$ (ml/kg/min)	24.63 (4.86)	24.75 (6.15)	− 0.11 (− 2.46, 2.22)	− 0.01	0.67 (0.29, 0.87)	3.22 (2.40, 4.91)	13.0	7.51 (30.4)
(n = 17)	$\dot{V}{\text{O}}_{2}$ (L/min)	1.74 (0.76)	1.75 (0.59)	− 0.01 (− 0.18, 0.16)	0.21	0.84 (0.60, 0.94)	0.24 (0.18, 0.36)	13.8	0.56 (32.2)
(n = 16)^*^	Heart rate (bpm)	119.31 (9.28)	120 (12.48)	− 0.69 (− 4.05, 2.67)	0.13	0.54 (0.11, 0.81)	9.80 (7.30, 14.92)	8.1	22.9 (18.9)
(n = 17)	RER	0.98 (0.06)	0.98 (0.06)	− 0.00 (− 0.03, 0.02)	− 0.01	0.57 (0.13, 0.82)	0.04 (0.03, 0.07)	4.1	0.09 (9.5)
RER = 1.00									
(n = 40)	$\dot{V}{\text{O}}_{2}$ (ml/kg/min)	19.88 (8.39)	19.83 (8.18)	0.05 (− 1.37, 1.46)	− 0.01	0.86 (0.75, 0.92)	3.56 (2.92, 4.55)	17.6	8.31 (41.1)
(n = 40)	$\dot{V}{\text{O}}_{2}$ (L/min)	1.45 (0.61)	1.52 (0.65)	− 0.06 (− 0.23, 0.10)	0.08	0.65 (0.43, 0.80)	0.40 (0.33, 0.51)	26.9	0.93 (62.9)
Log-transformed measurements									
2.0 W kg/FFM	Heart rate (bpm)	2.03 (0.01)	2.03 (0.01)	0.01 (− 0.01, 0.02)	**0.24**	–	–	–	–

CI: confidence interval; FFM: free fat mass; CV: coefficient of variation; ICC intraclass correlation; MDC: minimal detectable change; RER: respiratory exchange ratio SD: standard deviation; SEM: standard error measurement

*One outlier removed

**Two outliers removed

### Absolute and relative $\dot{V}{\text{O}}_{2}$

**Fig. 1:**
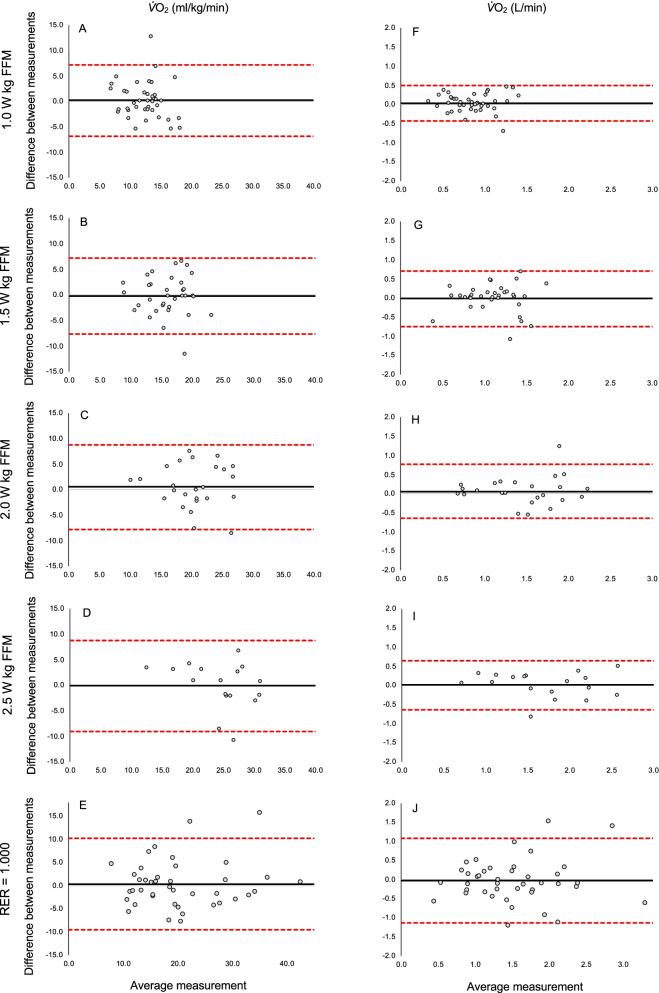
Bland–Altman plots representing the comparisons between baseline and 6 weeks for $\dot{V}{\text{O}}_{2}$ (ml/kg/min) and $\dot{V}{\text{O}}_{2}$ (L/min) at different workloads and when RER (respiratory exchange ratio) reached 1.00. The black horizontal line in each plot represents the mean difference between the two timepoints, with the upper and lower representing the limits of agreement (1 standard deviation)

Based on visual inspection of the plots in Fig. 1, little evidence of systematic error was observed. Except for relative $\dot{V}{\text{O}}_{2}$ assessed at 2.5 W kg/FMM when agreement reached excellent (ICC_2,1_ = 0.84), there was modest agreement between measures for absolute $\dot{V}{\text{O}}_{2}$ (ICC_2,1_ range 0.44 to 0.79) and relative $\dot{V}{\text{O}}_{2}$ (ICC_2,1_ range 0.64 to 74). The SEM% for absolute and relative $\dot{V}{\text{O}}_{2}$ ranged between 13.0% to 20.2%, and 13.8% to 26.3%, respectively_._ The MDC_90_% for absolute and relative $\dot{V}{\text{O}}_{2}$ ranged between 30.4% to 47.1%, and 32.2% to 61.4%, respectively. The lowest SEMs% and MDCs% for both absolute and relative $\dot{V}{\text{O}}_{2}$were observed for workloads at 2.5 W kg/FFM.

### Heart rate

**Fig. 2:**
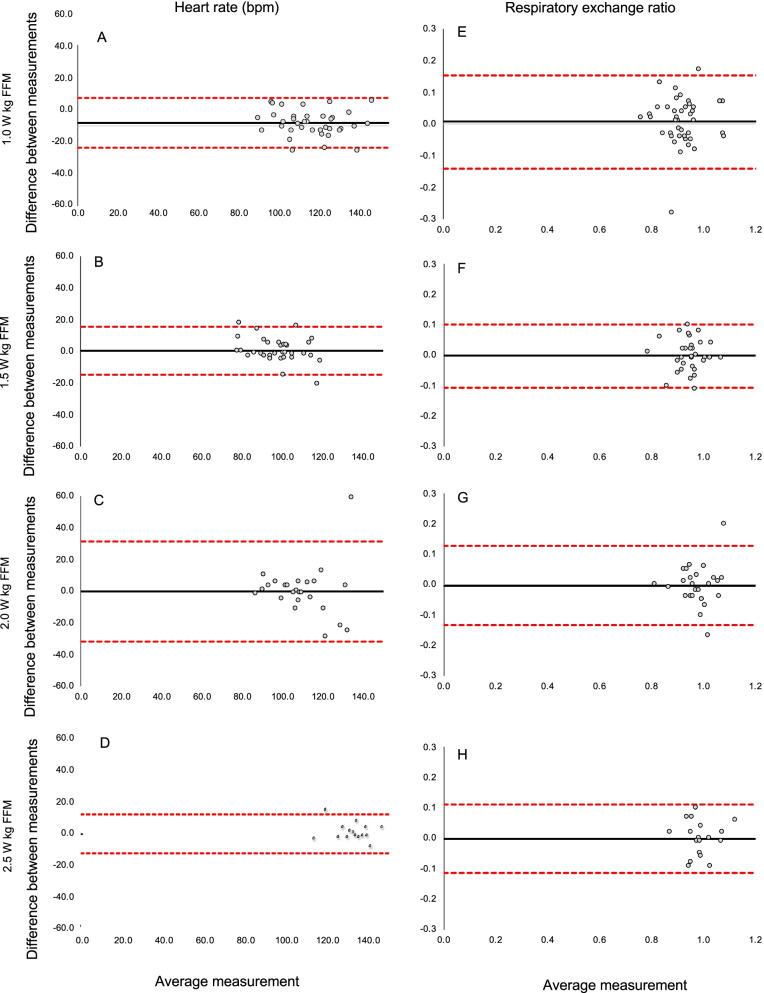
Bland–Altman plots representing the comparisons between baseline and 6 weeks for heart rate (bpm) and respiratory exchange ratios at different workloads and when RER (respiratory exchange ratio) reached 1.000. The black horizontal line in each plot represents the mean difference between the two timepoints, with the upper and lower representing the limits of agreement (1 standard deviation)

Based on visual inspection of the plots in Fig. 2A–C, little evidence of systematic error was observed. There was some evidence of heteroskedasticity for heart rate at workload 2.0 W kg/FFM, which was confirmed by correlation coefficient of 0.28, (*p* < 0.05). Hence, heart rate data at workload 2.0 W kg/FFM were log-transformed, but still heteroskedasticity remained (0.24, *p* < 0.05). There was modest agreement between measures for heart rate at workloads 1.0 W kg/FFM, and when RER = 1.000 (ICC_2,1_ range 0.57 to 0.65) with excellent agreement at workloads 1.5 W kg/FFM and 2.5 kg/FFM (ICC_2,1_ range 0.80 to 0.84). For heart rate, the SEM% ranged between 5.6% and 10.3%, and the MDC_90_% ranged between 13.0% and 24.1%.

### Respiratory exchange ratio

Based on visual inspection of the plots in Fig. 2D–F, little evidence of systematic variation was observed. There was modest agreement between measures for RER (range 0.57 to 0.65). For RER, the SEM% ranged between 3.2% and 5.4%, and the MDC_90_% ranged between 7.4% and 12.7%. The SEMs% and MDCs% were comparably low for all workloads assessed.

## Discussion

This study describes measures test–retest reliability of submaximal CRF at incremental workloads and when RER was equivalent to 1.000 in healthy active older adults. We found that relative reliability related to measures associated with submaximal CRF (absolute and relative $\dot{V}{\text{O}}_{2}$, heart rate and RER) was generally modest (ICC_2,1_ ≥ 0.44) according to previously described criteria [11]. Measurement error for heart rate and RER is arguably sufficiently low (≤ 10.3% SEM, ≤ 18.9 MDC_90_%) to detect real change across the workloads assessed. However, measurement errors in $\dot{V}{\text{O}}_{2}$ ranged up to 63% when RER was equivalent to 1.000, with an incremental decrease in measurement errors (SEM% and MDC_90_%) as workloads increased. The smallest measurement errors in $\dot{V}{\text{O}}_{2}$ were observed at the highest workload assessed (e.g. 2.5 W kg/FFM). Taken together, although at least modest relative reliability was consistently demonstrated, the smaller measurement errors associated with absolute and relative $\dot{V}{\text{O}}_{2}$ at 2.5 W kg/FFM may indirectly suggest that examining submaximal CRF at higher workloads is more useful to monitor changes more confidently.

Some studies have defined a non-response in CRF ($\dot{V}{\text{O}}_{{2\,{\text{peak}}}}$) in older adults (> 50 years) as a change < 0 L/min [12], others as less than 5% [13] or 204 mL/min [14]. Other research has used day-to-day variability within participant coefficient of variation of 5.6% to define $\dot{V}{\text{O}}_{2}$ response [15]. However, data on $\dot{V}{\text{O}}_{{2\,{\text{max}}}}$ or $\dot{V}{\text{O}}_{{2\,{\text{peak}}}}$ may have limited practical application to older adults. Many older populations, including the active ageing population, cannot undergo maximal exercise testing to volitional exhaustion (e.g. capability and/or safety). Our findings suggest that much larger changes than previously used in the literature for peak CRF are required to ensure that change in $\dot{V}{\text{O}}_{2}$ submaximal in response to a treatment exceeds possible measurement error. The MDC is the minimum amount of change in a measure unlikely to be due to chance variation in measurement and is interpreted as the minimum amount of change required to designate the change as real and beyond the bounds of measurement error. We computed the MDC at the 90% confidence interval, and the interpretation of MDC_90_ is that 90% of truly stable individuals will display random variation on subsequent testing equal to or less than the MDC_90_ value. Therefore, our data suggest that if changes in submaximal CRF do not exceed the MDC_90_, the assessor cannot be confident that observed changes are beyond measurement error associated with the testing protocol evaluated in the current study. As workload increased, the measurement error appeared to lower such that our maximum workload was associated with the lowest MDC_90_ of ~ 30%. These data suggest $\dot{V}{\text{O}}_{2 submax}$_2_ beyond 30% ensures real change at an individual level. Given that detecting a 30% change may be difficult in practice, researchers and health professionals may opt to focus on measures other than submaximal fitness, such as healthy behaviours (regular physical activity) for healthy individuals.

A strength of this study is the assessment of minutes spent in physical activity in the 6 weeks prior to the 6-week study period, where the time in physical activity remain unchanged. In addition, we implemented standard assessment procedures taking into account relative workload (W/kg), body composition (i.e. fat free mass and fat mass) that may influence power output. Another strength of our study is the utility of our results to two different approaches of determining submaximal CRF. Namely, submaximal CRF can be extracted based on $\dot{V}{\text{O}}_{2}$ at the relative workload and supported by heart rate and RPE data [2]. Alternatively, submaximal $\dot{V}{\text{O}}_{2}$ can be determined at the point where the RER reaches 1.000, which is considered the aerobic-to-anaerobic crossover [2]. Notably, the relatively large measurement error associated with the latter option may influence researchers and/or clinicians towards the former approach.

There are several limitations to our study that warrant consideration. First is the arbitrarily chosen level of statistical significance (*p* < 0.05) level to discern the presence or absence of heteroskedasticity. In the absence of well-established cut-off points correlation coefficients, this was done to create objectivity regarding whether data were heteroskedastic or not. It is possible that interpretation of our results would differ if different criteria were used to discern the presence or absence of heteroskedasticity. Similarly, different ICC cut-offs have been used in the clinical literature to determine the agreement between measures. Second, participants in the current study were limited to 17 active older adults at higher work loads. Hence, we caution generalising our finding to other samples (e.g. free-living sedentary, pathological) and recommend clinimetric evaluation in adequately powered studies. Third, time in very vigorous activity per day changed by 4 min (*p* = 0.05), which may influence our findings, given that intensity modulates fitness. Fourth, our data do not provide information about the minimal clinically important difference. Although we determined the MDC, which tells assessors the amount of change needed to be sure of *real* change beyond that associated with measurement error, it not necessarily the same as the minimal clinical importance difference. Future research is needed to determine the minimal clinical importance difference so that researchers and clinicians can determine the amount of change in submaximal CRF is required with interventions to achieve meaningful clinical improvements in health status for the patient.

## Conclusions

In summary, at least modest relative reliability was consistently demonstrated. Measurement error for absolute and relative $\dot{V}{\text{O}}_{2}$ improved as workload increased, with largest measurement errors found when RER was equivalent to 1.000. The smaller measurement errors associated with absolute and relative $\dot{V}{\text{O}}_{2}$ at 2.5 W kg/FFM may indirectly suggest that examining submaximal CRF at higher workloads is more useful to monitor changes more confidently.

## Supplementary Information

**Additional file 1**. Time in physical activity 6-week during and 6-week prior to first cardiorespiratory fitness assessment.

## Data Availability

The datasets used and/or analysed during the current study are available from the corresponding author on reasonable request.

## Abbreviations

CRF: Cardiorespiratory fitness
FFM: Free fat mass
ICC: Intraclass correlation coefficient
MDC: Minimal detectable change
RER: Respiratory exchange ratio
SEM: Standard error of measurement
W: Watts

## References

[1] Harber MP, Kaminsky LA, Arena R, Blair SN, Franklin BA, Myers J (2017). Impact of cardiorespiratory fitness on all-cause and disease-specific mortality: advances since 2009. Prog Cardiovasc Dis.

[2] Winter EM, Davison RC, Bromley PD, Mercer T. Sport and exericse physiology testing guidelines: volume II—exercise and clinical testing The British Associated of Sport and Exercise Sciences Guide. Routledge. 2009

[3] Noonan V, Dean E (2000). Submaximal exercise testing: clinical application and interpretation. Phys Ther.

[4] Atkinson G, Nevill AM (1998). Statistical methods for assessing measurement error (reliability) in variables relevant to sports medicine. Sports Med.

[5] Bland JM, Altman DG (1986). Statistical methods for assessing agreement between two methods of clinical measurement. Lancet.

[6] Stratford PW, Goldsmith CH (1997). Use of the standard error as a reliability index of interest: an applied example using elbow flexor strength data. Phys Ther.

[7] Stratford PW. Getting more the literature: estimating the standard error of measurement from reliability studies. Physiotherapy Canada 2004;56:27–30.

[8] Ross R, Goodpaster BH, Koch LG, Sarzynski MA, Kohrt WM, Johannsen NM (2019). Precision exercise medicine: understanding exercise response variability. BJSM.

[9] Huschtscha Z, Parr A, Porter J, Costa RJS (2021). The effects of a high-protein dairy milk beverage with or without progressive resistance training on fat-free mass, skeletal muscle strength and power, and functional performance in healthy active older adults: a 12-week randomized controlled trial. Front Nutr.

[10] Borg GA (1982). Psychophysical bases of perceived exertion. Med Sci Sports Exerc.

[11] Fleiss J (1986). The design and analysis of clincial experiements.

[12] Chmelo EA, Crotts CI, Newman JC, Brinkley TE, Lyles MF, Leng X (2015). Heterogeneity of physical function responses to exercise training in older adults. J Am Geriatr Soc.

[13] Pandey A, Swift DL, McGuire DK, Ayers CR, Neeland IJ, Blair SN (2015). Metabolic effects of exercise training among fitness-nonresponsive patients with type 2 diabetes: the HART-D Study. Diabetes Care.

[14] Ross R, de Lannoy L, Stotz PJ (2015). Separate effects of intensity and amount of exercise on interindividual cardiorespiratory fitness response. Mayo Clin Proc.

[15] Dalleck L, Haney DE, Buchanan CA, Weatherwax R (2016). Does a personalised exercise prescription enhance training efficacy and limit training unresponsiveness? A randomised controlled trial. J Fit Res.

