# Supplementary material for: What is real change in submaximal cardiorespiratory fitness in older adults? Retrospective analysis of a clinical trial
Source: Sports Med Open. 2022 Apr 28;8:59. doi: 10.1186/s40798-022-00447-6 (PMC9051008; doi:10.1186/s40798-022-00447-6)
Supplement: Supplementary file 1 — Additional file 1. Time in physical activity 6-week during and 6-week prior to first cardiorespiratory fitness assessment. [file 40798_2022_447_MOESM1_ESM.docx]

Article Title: What is real change in sub-maximal cardiorespiratory fitness in older adults? Retrospective analysis of a clinical trial

Journal: Sports Medicine - Open

Author names: Michelle Hall, Yuri Lopes Lima, Zoya Huschtscha, Fiona Dobson, Ricardo J.S. Costa

Affiliation and email address of the corresponding author: halm@unimelb.edu.au | Centre for Health, Exercise and Sports Medicine, Department of Physiotherapy, School of Health Sciences, Melbourne, The University of Melbourne, VIC, Australia

| **Supplementary Table** Time in physical activity 6-week during and 6-week prior to first cardiorespiratory fitness assessment | | | | | |
| --- | --- | --- | --- | --- | --- |
|  | 6-week interval prior first cardiorespiratory fitness assessment  n=41 | 6-week interval post first cardiorespiratory fitness assessment  n=41 | Mean difference (95% confidence interval) | P-Value |  |
| Minutes in light intensity activity per day | 310 (144) | 308 (160) | 1 (-8, 10) | 0.80 |  |
| Minutes in moderate intensity activity per day | 201 (61) | 202 (61) | -1 (-10, 6) | 0.70 |  |
| Minutes in vigorous activity per day | 18 (24) | 22 (20) | -4 (-11, 2) | 0.15 |  |
| Minutes in very vigorous activity per day | 8 (13) | 12 (14) | -4 (-8, 0) | 0.05 |  |
